# Supplementary material for: High Expression of a tRNAPro Derivative Associates with Poor Survival and Independently Predicts Colorectal Cancer Recurrence
Source: Biomedicines. 2022 May 12;10(5):1120. doi: 10.3390/biomedicines10051120 (PMC9138872; doi:10.3390/biomedicines10051120)
Supplement: Supplementary file 1 [file biomedicines-10-01120-s001.zip › biomedicines-1697955-supplementary/Supplementary Tables/Table S1.pdf]

**Table S1.** List of qPCR primers used in the current study.

| Primer name | Primer sequence (5′→3′) | Length (nt) | $T_m$ (°C) |
|---|---|---|---|
| 5′-tiRNA-Pro$^{TGG}$ forward primer | GGTCTAGGGGTATGATTCTCGGTT | 24 | 61.2 |
| *RNU48* forward primer | TGATGATGACCCCAGGTAACTCT | 23 | 60.6 |
| *RNU43* forward primer | ACTTATTGACGGGCGGACA | 19 | 59.0 |
| Universal reverse primer | GCGAGCACAGAATTAATACGAC | 22 | 57.9 |
