# Supplementary material for: High Expression of a tRNAPro Derivative Associates with Poor Survival and Independently Predicts Colorectal Cancer Recurrence
Source: Biomedicines. 2022 May 12;10(5):1120. doi: 10.3390/biomedicines10051120 (PMC9138872; doi:10.3390/biomedicines10051120)
Supplement: Supplementary file 1 [file biomedicines-10-01120-s001.zip › biomedicines-1697955-supplementary/Supplementary Tables/Table S2.pdf]

Table S2. Associations between 5′-tiRNA-Pro^{TGG} expression status and the most significant clinicopathological parameters of colorectal adenocarcinoma patients.

Covariate	5′-tiRNA-Pro^{TGG} expression status		
	**Negative**	**Positive**	***P* value**
**Gender**			0.39
Male (n=82)	60 (73.2%)	22 (26.8%)	
Female (n=73)	51 (69.9%)	22 (30.1%)	
**Tumor site**			0.053
Colon (n=107)	82 (76.6%)	25 (23.4%)	
Rectum (n=48)	29 (60.4%)	19 (39.6%)	
**Histological grade**			0.066
I (n=13)	12 (92.3%)	1 (7.7%)	
II (n=115)	82 (71.3%)	33 (28.7%)	
III (n=27)	17 (63%)	10 (37%)	
**T**			0.65
T1 (n=3)	3 (100%)	0 (0%)	
T2 (n=17)	12 (70.6%)	15 (29.4%)	
T3 (n=96)	66 (68.8%)	30 (31.3%)	
T4 (n=39)	30 (76.9%)	9 (23.1%)	
**N**			0.19
N0 (n=85)	64 (75.3%)	21 (24.7%)	
N1 (n=39)	28 (71.8%)	11 (28.2%)	
N2 (n=31)	19 (61.3%)	12 (38.7%)	
**M**			0.56
M0 (n=138)	99 (71.7%)	39 (28.3%)	
M1 (n=17)	12 (70.6%)	5 (29.4%)	
**TNM stage**			0.44
I (n=17)	13 (76.5%)	4 (23.5%)	
II (n=65)	48 (73.8%)	17 (26.2%)	
III (n=56)	38 (67.9%)	18 (32.1%)	
IV (n=17)	12 (70.6%)	5 (29.4%)	
