# Supplementary figures and images for: High Expression of a tRNAPro Derivative Associates with Poor Survival and Independently Predicts Colorectal Cancer Recurrence
Source: Biomedicines. 2022 May 12;10(5):1120. doi: 10.3390/biomedicines10051120 (PMC9138872; doi:10.3390/biomedicines10051120)

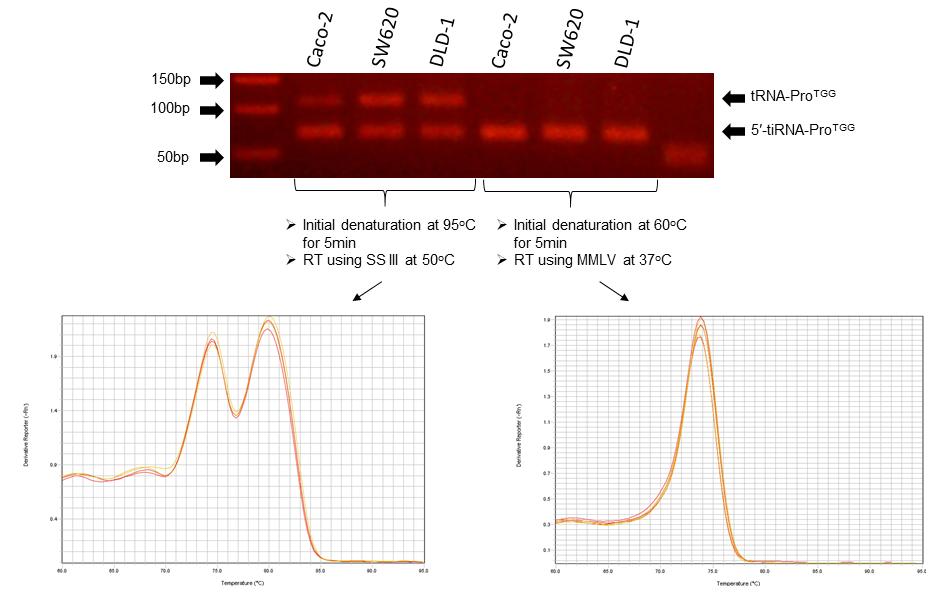

Supplement: Supplementary file 1 [file biomedicines-10-01120-s001.zip › biomedicines-1697955-supplementary/Supplementary Figures/Figure S1.tif]

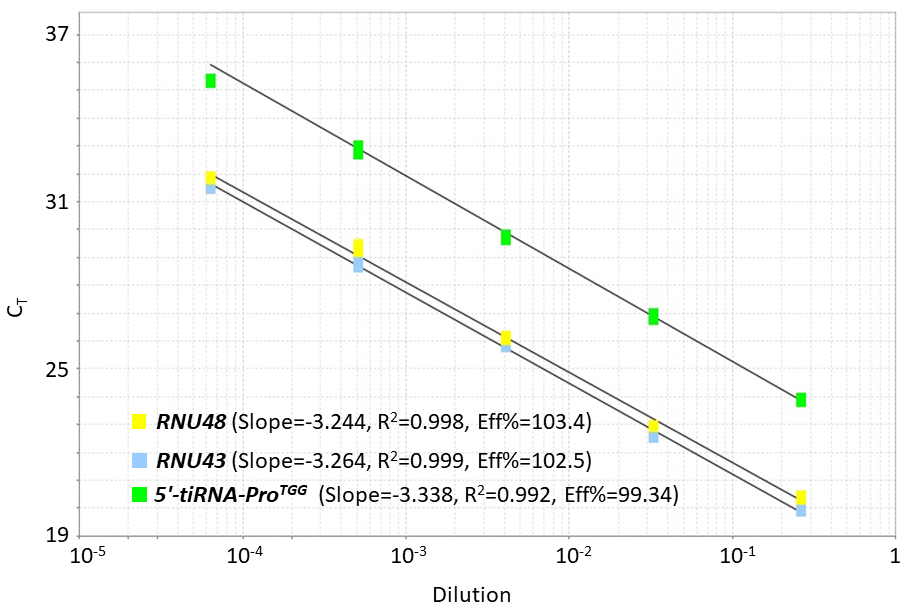

Supplement: Supplementary file 1 [file biomedicines-10-01120-s001.zip › biomedicines-1697955-supplementary/Supplementary Figures/Figure S2.tif]

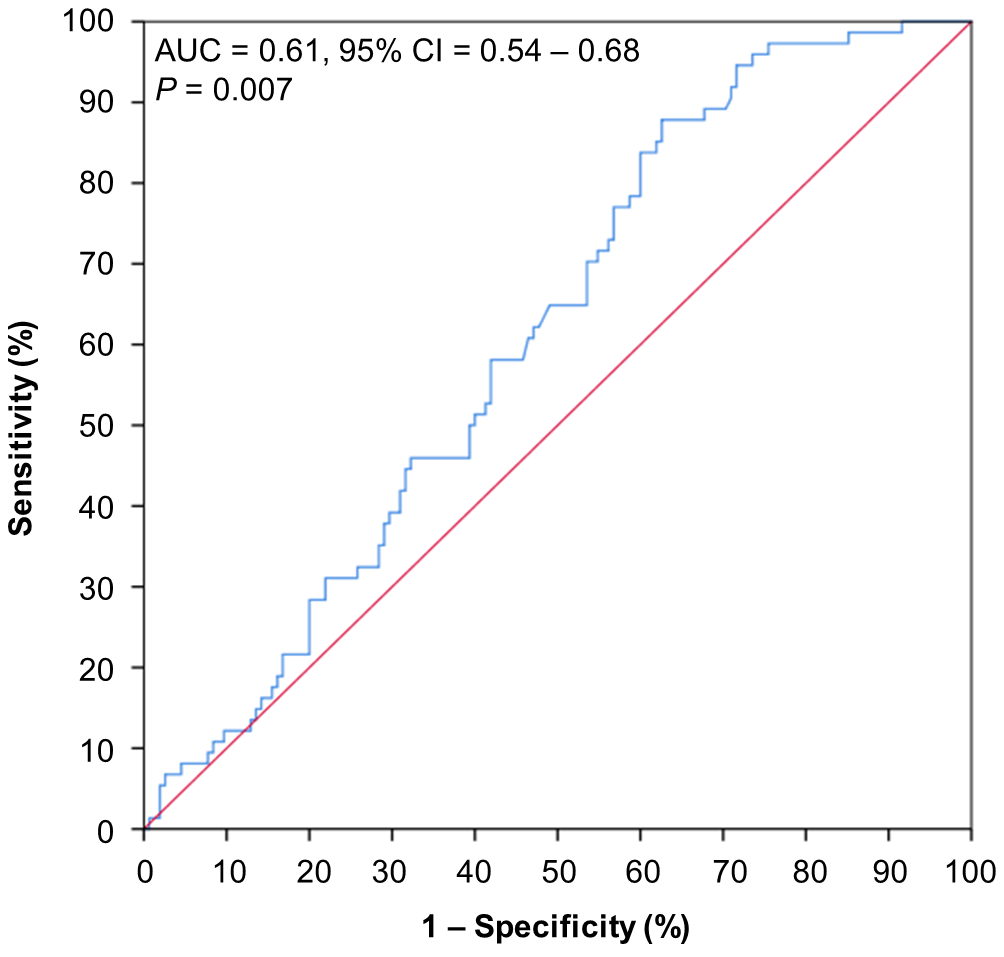

Supplement: Supplementary file 1 [file biomedicines-10-01120-s001.zip › biomedicines-1697955-supplementary/Supplementary Figures/Figure S3.tif]

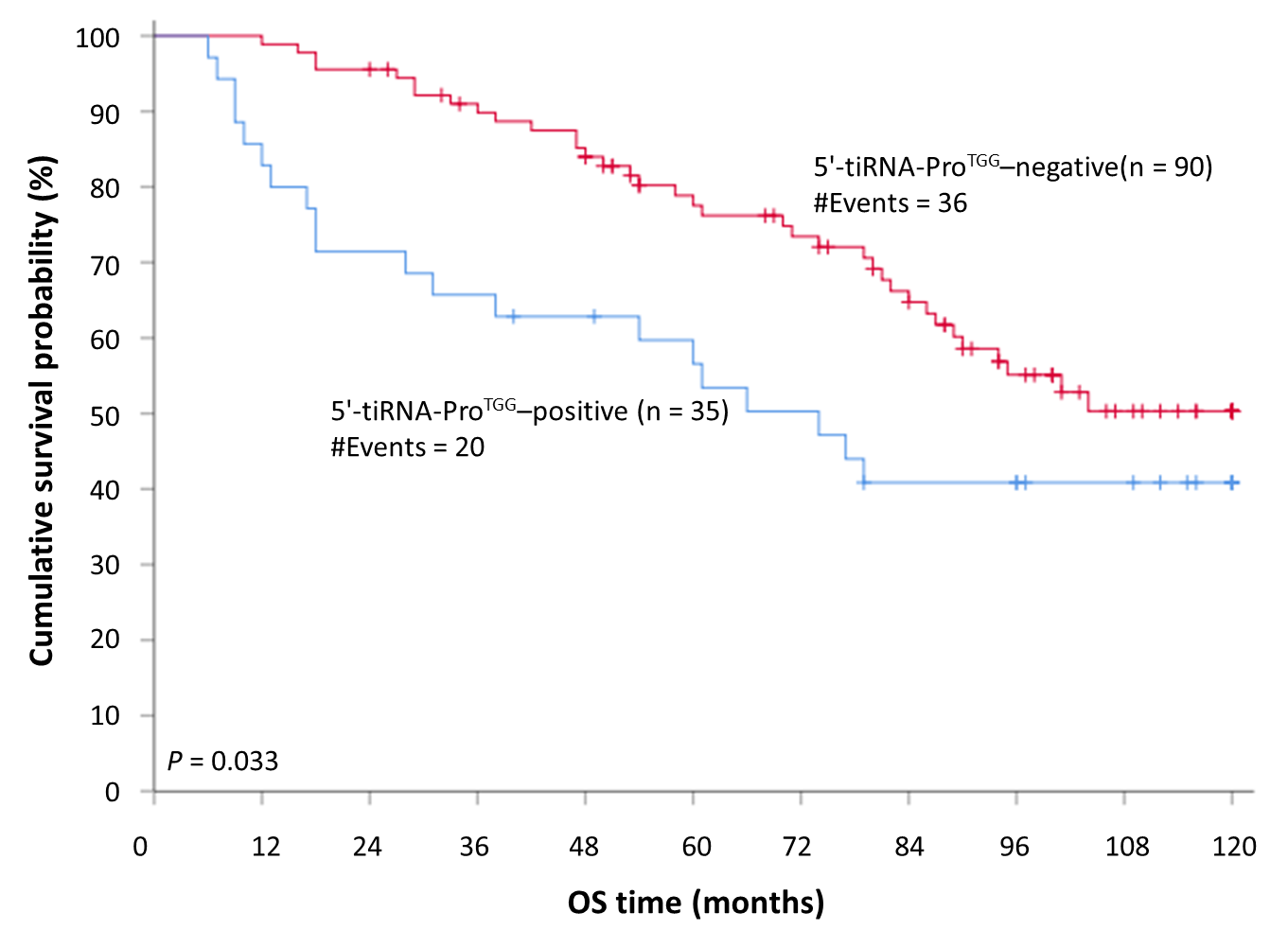

Supplement: Supplementary file 1 [file biomedicines-10-01120-s001.zip › biomedicines-1697955-supplementary/Supplementary Figures/Figure S4.tif]
